# The diagnostic role of emotions in feminist philosophy

**DOI:** 10.3389/fpsyg.2025.1622438

**Published:** 2025-08-12

**Authors:** Valentina Bortolami

**Affiliations:** Department of Philosophy, Sociology, Education and Applied Psychology (FISPPA), University of Padua, Padua, Italy

**Keywords:** emotions, feminist phenomenology, feminist epistemology, oppression, emancipation

## Abstract

The article explores feminist phenomenological and epistemological literature to investigate the meaning of the double ontological shock, i.e., the experience of intuiting that reality differs from appearances without being able to clearly articulate what is really happening, which is relevant not only for feminists but also more generally for those who wish to reflect on the diagnostic role of emotions. The analysis of the double ontological shock supports the idea that emotions are a primary source of motivation and action orientation. This is particularly relevant in relation to situations of oppression, where emotions clearly express their diagnostic and world-disclosing nature.

## 1 Introduction

Emotions^1^ have been understood for centuries in opposition to rationality, that is, as obstacles to properly understanding reality. However, there is now a “near-consensus” that they play an important role in knowledge processes, decision making, motivation, and moral reasoning (Tappolet et al., 2011, p. 2). This article renegotiates the capacity of emotions in light of this idea by exploring the feminist case of *double ontological shock* (Bartky, 1975, p. 434), which relates to the *thorny problem of the emancipation of the oppressed individual* (Daigle, 2014, p. 207). Both are relevant for feminist concerns regarding situations in which external criteria seem to be inadequate for assessing the lived experience of the subjects, opening up reflection on the role of emotions, not only in understanding what happens, but also diagnosing what is wrong in a given situation.

This analysis aims to provide insights on the general problem of diagnosis by approaching it from a specific set of concerns and methodologies belonging to feminist theory. Indeed, feminist theory can be understood as a struggle to diagnose oppressive features of the world (those that Alia Al-Saji calls *pathologies of the social*^2^) and to identify appropriate ‘therapy’. Furthermore, feminist theory can help elucidate different emotional dimensions, namely their embodied and situated nature, their orientation toward action, their inherent relationality, their capacity to transcend oppressive structures, and their role in diagnosis. More specifically, this article employs and develops ideas that emerge from feminist standpoint theory.^3^

Emotions provide a special kind of information. Indeed, one prominent characteristic, or the ‘essence’ of emotions (Slaby, 2014, p. 32), seems to be their ability to inform us of what matters to us.^4^ This facet of emotions has been explored by feminist epistemologies (among other disciplines), which makes a case for emotions as evidence or guides in our knowledge processes (as values and emotions are connected, Jaggar, 1989; Anderson, 2004) and investigates the role of interpersonal and collective activities in these processes (Jaggar, 1989; Candiotto, 2023). Feminist methodology also emphasizes the role of emotions in research, recommending, for example, that we seriously consider “mental or bodily experiences, including *our emotions*, as they are tools for conducting affective research on sensitive topics (Carroll, 2013, p. 558)” (Leavy and Harris 2018, emphasis mine).

This article provides three theses on emotions. The first thesis regards methodology: emotions should be studied with the tools of phenomenology *and* epistemology. The second thesis regards the relevance of the feminist case of double ontological shock, the experience of intuiting that reality differs from appearances without being able to clearly articulate what is truly happening, maintaining its interest not only for feminists, but also, more generally, for those who aim to reflect on the diagnostic role of emotions. Consequently, the article explores the feminist phenomenological and epistemological literature to inquire into the meaning of double ontological shock. The third thesis regards the ontology of emotions. As will be shown in the analysis of double ontological shock, emotions are a primary source of motivation and action-orientation. This is particularly relevant concerning situations of oppression, in which emotions express clearly their diagnostic and world-disclosing nature.

## 2 Methodology

Emotions are an elusive object of inquiry, defying dualisms established in western thought. As Rebekka Hufendiek states: ‘*Emotions are notoriously difficult to categorize, and they seem to cross borders between categories that philosophers traditionally have wanted to separate, like body and mind, nature and culture, rationality and irrationality*’ (Hufendiek, 2016, p. 3). This observation proves even more relevant from a feminist perspective, because the dualisms listed by Hufendiek are embedded in a gendered framework. Body, nature, and irrationality are attributed to women or considered “feminine” traits viewed as inferior to such masculine traits as mind, culture, and rationality (Lloyd, 1979; Jaggar, 1989). For this reason, feminist thinkers are often wary of such dualisms (body and mind, nature and culture, rationality and irrationality), and have criticized them since the beginning of women’s participation in science and research (that is, in the West, since the early twentieth century when education became accessible to women). For example, even before the ‘affective turn’ (Gorton, 2007, p. 333) feminist philosophers have advocated for a reconsideration of Reason (Lloyd, 1979; Jaggar, 1989) and a recognition of the important role emotions play in knowledge processes. Understanding emotions not as mere agents of irrationality but as contributors to knowledge processes, stands in continuity with this work of critique and the production of alternative interpretations and research, disrupting the traditional dualisms that implicitly operate in thinking about emotions. Moreover, this conception reconfigures the question of how to properly investigate emotions, if only to make room for their epistemological dimension, that is, understanding the methodological consequences that arise when emotion challenges dualisms.

Notably, from a methodological perspective, questioning the fundamental nature-culture dualism influences the chosen methodology for research in emotion. Determining whether emotions are natural or cultural, in fact, narrows down which disciplines are best-suited to make this inquiry. If they are cultural, the appropriate choice would be disciplines such as anthropology, (social) psychology, philosophy; if they are natural, the disciplines of neuroscience, biology, neurology make more sense.

The reality is that emotions do not lend themselves to a categorization of traditional dualisms. The most convincing hypothesis is that they are complex phenomena that transcend these distinctions, simultaneously natural and cultural—in the mind and the body, rational and irrational—thus requiring an interdisciplinary approach for their comprehension. In philosophy, the methodological consequences of an anti-dualist reconsideration of emotions pertain to the fact that they can be approached by multiple perspectives linked to different philosophical traditions. The most notable and relevant are the naturalized framework and the phenomenological framework.

Contemporary philosophical research on emotions is often naturalized, pursuing philosophical inquiry in close relation to empirical research in psychology, neuroscience, and the health sciences, thus privileging the ‘science and nature’ side of the aforementioned dualisms.^5^

A less traditional naturalized approach, which has gained interest in contemporary debates, is that offered by feminist epistemologies and new materialisms, that embrace a philosophical naturalism particularly interesting for the study of emotions. This kind of naturalism is anti-reductionist and anti-dualist and does not privilege the natural sciences over the historical and social sciences.^6^ More notably from a phenomenological perspective, various naturalized feminist epistemologies consider the distinction between subject and object, and the role of the subject in acquiring scientific knowledge, in a fashion that is very distinctive and radically departs from other naturalisms, as it does not separate the knowing subject(s) from the object they know and the context, that is, the reality in which they are embedded.^7^

Nevertheless, since reconfiguring research in a non-dualistic fashion is an extremely hard venture, naturalized approaches still may lead to overestimating scientific approaches while underestimating the real, lived experiences of the subjects involved (subjects that are considered ontologically entangled with the object of the knowledge and their context by feminist epistemology). Therefore, adopting a naturalized philosophical approach, even if sophisticated and anti-reductionist as it occurs in feminist epistemology and new materialism (or in Hufendiek’s theory of emotions), can present some problems, including the one most relevant to this study, namely, the tendency to neglect the experiences and contexts that precede the phenomenon of knowledge in the strict sense.

Phenomenology can be (a part of) the solution: both the phenomenological approach itself, and the resources of phenomenological philosophical literature, indeed, may help counterbalance the naturalized aspect of an inquiry into emotions. This is particularly relevant with respect to the inquiry into emotions connected with experiences of oppression, because phenomenology also allows the exploration of oppression, the related idea of the resistance of the oppressed, and finally, the understanding of the role of negative and painful emotions. So, even if epistemology is necessary to comprehensively understand the role of emotions in knowledge processes, phenomenology must also be integrated. Phenomenology helps to question the presupposition that the study of emotions should be executed by the sciences. Furthermore, it engages in a deeper and more robust manner with the phenomenon, capturing aspects of the experience of subjects (particularly those who are part of oppressed and marginalized communities) that would be underestimated by relying only on a ‘scientific’ understanding of this relationship (no matter how anti-reductionist).

These epistemological attempts to approach emotions have metaphysical consequences as well that lead to the appreciation of relational ontologies (which cannot be fully addressed in this article, but which are briefly discussed in the conclusions). To conclude the discussion of methodology, consider also that even the most naturalized understanding of emotions cannot avoid confronting the “phenomenology of emotions.” This does not mean that naturalized approaches utilize a phenomenological method, as the latter is not only concerned with the lived experience but also with the structures that inform it and that ‘makes a difference in’ it (Al-Saji, 2017, p.149);^8^ instead, it means that without their phenomenological aspect, emotions would not be emotions at all. Without considering them as lived experience, we would be analyzing something different.

## 3 Emotion and diagnosis in the oppression-related contexts

### 3.1 The thorny problem

The tension between being oppressed and being able to recognize it—diagnose it—is crucial to any kind of anti-oppressive thinking, even when it is not made explicit. The issue is not only phenomenological but also epistemological (and metaphysical^9^). In fact, on the epistemological level this problem raises the question of how we can trust ourselves to be authoritative about our experience on the one hand and about reality (or in other terms, about ‘the world’) on the other. In Christine Daigle’s words, it is the ‘thorny problem of thinking through the emancipation of the oppressed individual*’* (which I will refer to from now on as ‘the thorny problem of emancipation’):

*How does the oppressed, who does not see herself as such and perhaps contributes to her own oppression, precisely because she has internalized the patterns of oppression, come to the necessary realization? How does an oppressive and alienating system come to be acknowledged as such so that the situation that is made for the individual may begin to change?* (Daigle, 2014, p. 207).

This “thorny problem” identifies a tension between an intuition about being oppressed and external criteria of judgment that prove inadequate, failing to account for the subject’s intuitive perceptions. In this discrepancy between the available categories for understanding reality and lived experience lies the space to develop hope for change. Two examples related to the gender dimension and the historical oppression of women illustrate this problem and facilitate an understanding of its importance.^10^

The first example concerns women as epistemic authorities. Let us say that one has been taught that she cannot be a rational person because she is a woman. Despite this, at a certain point she *feels* that her experience clashes with this teaching. Nevertheless, because she learned that she cannot believe, or trust, her own thoughts and intuition (which are supposedly the thoughts and intuition of a non-rational person), she finds herself experiencing self-doubt and uncertainty (the double ontological shock described by Bartky, which I will expand on momentarily). What happens next is particularly interesting for epistemology and reflection on the role of emotions in knowledge processes, and it may depend on a multiplicity of variables (as detailed below). This can lead to a redefinition of what being rational means and, or, to the redefinition of the status quo via social change processes.

A second example is sexual violence in the context of existing romantic relationships. A woman may have been taught that rape cannot occur in a romantic relationship. If her partner forces her to engage in unwanted sexual activity, she will perceive the experience as unpleasant, negative and unfair, even though she lacks the conceptual categories to define it as violence. How do we explain this negative emotional response to something that is not considered wrong on a social level, or even by the subject herself? This example challenges the cognitivist understanding of emotions and raises the epistemological question of how it has been possible to conceive and expand the concept of rape in a society that initially lacked or severely restricted such a concept.

To generalize even more, the problem that emerges from both examples is to understand how it is possible in a society in which one is oppressed and, therefore, taught oppressive beliefs about oneself (and about other people), to understand the same reality differently (to use Bartky’s words), and therefore produce (not just utilize pre-existing concepts, but *produce*) concepts like oppression, rape, white supremacy, racism, etc.

### 3.2 Double ontological shock and outlaw emotions

Sandra Lee Bartky provides us with a useful name for the experience exemplified in the cases above, that is “double ontological shock”:

*In sum, feminists suffer what might be called a «double ontological shock»: first, the realization that what is really happening is quite different from what it appears to be happening, and, second, the frequent inability to tell what is really happening at all* (Bartky, 1975, p. 434).

Bartky’s phenomenological perspective focuses on victimization, in this case of women,^11^ describing the ways in which victimization and oppression can have a grip on their psychology. Double ontological shock is the name given to the experience of ‘knowing’ (but that, I am arguing here, may be also described as ‘feeling’) that what is happening is not what appears to be happening, even if one is unsure about what is actually happening. Various circumstances can contribute to the ‘inability to tell what is really happening at all’, the most notable of which is the impossibility, for subjects, of believing that they can understand their own situation and judge it appropriately. For Bartky (1990, pp. 29–30), this difficulty of believing that the oppressed can indeed understand reality is the result of a process of dehumanization and depersonalization. Double ontological shock is an effect of psychological oppression, but also the beginnings of an insight that can bring the subject to an enhanced understanding of reality, that is, it contains the seed to produce new knowledge. It can be understood as a kind of partial insight: it is the knowing that something is wrong, but it is a partial knowing that has yet to conclude what is *really* happening, what is the *real* situation. Precisely because of this dehumanization and depersonalization, the case of double ontological shock is an extreme one, in which emotions that signal that something is wrong work as symptoms that are not immediately understood by the subject, who has been accustomed to disbelieving their own symptoms or being subject to disbelief by other people.

In the article “Love and Knowledge” (1989), Alison Jaggar describes and analyzes the experience of uncertainty, which we have named *double ontological shock* and *thorny problem of emancipation*.^12^ Jaggar’s perspective focuses on the epistemological relevance of emotions and on the gains, in terms of knowledge, of the oppressed. The idea that oppressed people can obtain a gain, or “epistemic privilege” is maintained by feminist standpoint theory and is related to the importance of collectively addressing double ontological shock. Jaggar describes double ontological shock or the moment in which the subject *feels-and-knows* something is wrong, but doubts herself, as follows:

When unconventional emotional responses are experienced by isolated individuals, those concerned may be confused, unable to name their experience; they may even doubt their own sanity*. Women may come to believe that they are ‘emotionally disturbed’ and that the embarrassment or fear aroused in them by male sexual innuendo is prudery or paranoia* (Jaggar, 1989, p. 166, my emphasis).

Both Bartky and Jaggar maintain that there is first and foremost a problem of friction between the emotional life of the subject and the external or imposed description that express confusion and an inability to name the problem—to understand what is happening—nevertheless retain the awareness that something is indeed wrong. In these moments, the woman (or marginalized other) questions her own sanity, because she has been raised in a society that does not allow her to immediately believe what she feels. These “unconventional emotional responses” are referred to as “outlaw emotions” by Jaggar and describe a friction or rupture that occurs between emotions and external norms, between inner experience and the world. Like physical symptoms that signal bodily dysfunction before a medical diagnosis is reached, outlaw emotions serve as indicators that something is amiss in the social fabric, even when explicit social critique has yet to be formulated.

To recap, double ontological shock is an experience of uncertainty that tells us something is wrong without knowing what to do with that information, while the outlaw emotions are the emotions related to that experience. Outlaw emotions may be understood as a perception of symptoms that is already the beginning of a process of diagnosis. However, on their own, they cannot bring about change, even though they are necessary for change. For the feminists cited, it is necessary to proceed with the construction of a consciousness and/or subculture.

### 3.3 Collectively building a way out

Outlaw emotions experienced in double ontological shock signal that something is wrong. But in themselves, they cannot actually diagnose the ‘pathologies of the social’: double ontological shock and its related emotions constitute only a *partial* insight. To ascertain a proper insight, it is necessary to build a “feminist consciousness” (Bartky, 1975) or “subculture” and “standpoint” (Jaggar, 1989). In both cases, the step that follows double ontological shock is the construction of an alternative understanding of the world. Jaggar explains this step as follows:

*When certain emotions are shared or validated by others, however, the basis exists for forming a subculture defined by perceptions, norms, and values that systematically oppose the prevailing perceptions, norms, and values. By constituting the basis for such a subculture, outlaw emotions may be politically because epistemologically subversive* (Jaggar, 1989, p. 166).

Within relationships with other people, it is possible to collectively build conceptual tools (“perceptions, norms, and values”) that allow another interpretation of an experience relating to the recognition of oppression that ultimately leads to the creation of a subculture. But this does not solve – in itself – the thorny problem; it only moves the problem from the individual level to the collective level. In other words, the thorny problem has merely been translated; the question of how it is possible, for the collective, to create this alternative space for the construction of new and subversive ‘perceptions, norms, and values’ in opposition with mainstream culture, despite the conditioning suffered by oppressed people, remains. Moreover, the issue of how double ontological shock can be better addressed collectively rather than individually remains open.

Intuitively, the notion that a collective understanding of the world is better than an individual understanding sounds reasonable. Nonetheless, the immediacy of this intuition does not explain or justify why it is so. However, it is important to note that this notion has been confirmed by research in social psychology (Candiotto, 2023, p. 912) and corroborated also by the history of feminist theory and research. A notable example offered by Mulinari and Sandell (1999) is that consciousness-raising groups have established feminist scientific research.

The importance of a collective understanding of the world is highlighted in feminist standpoint theory (Hartsock, 1983; Harding, 1986, 1993). This theory can be summarized in three fundamental theses: the positioning thesis (or the “situated knowledge” thesis^13^), the epistemic privilege thesis, and the achievement thesis (Toole, 2019). The positioning thesis concerns the fact that there are oppressed people. In Bartky’s terms, they are ‘victimized’ and therefore experience double ontological shock. The thesis of epistemic privilege states that precisely because of their position of oppression, the oppressed are capable of a more realistic view of social relations than their oppressors. One of the reasons for this epistemic privilege is the ‘dialectical’ nature of their position, i.e., the fact that marginalized groups cannot take reality at face value, but are more inclined, due to their position in respect of oppression, to notice the power relations and the structures of oppression informing society. However, this epistemic privilege is not prerogative of oppressed *individuals* but can be achieved through a process of *collective* knowledge production. The achievement thesis refers to this processual and collective aspect of the “privileged” knowledge acquired by oppressed people.

Bartky focuses primarily on the aspect of psychological suffering, however, standpoint theory reflects on the moment of uncertainty illustrated by Bartky and Jaggar, and explains its advantageous epistemological characteristics, (Jaggar also explicitly refers to standpoint theory in search of criteria for assessing which outlaw emotions should be used to understand the world and which should not). Furthermore, standpoint theory reflects on the need for a collective process of elaborating the experience of the oppressed, as Bartky and Jaggar do. In this process, there must be something special that can explain why a change in understanding the world can be articulated in the collective dimension.

One element that can help to explain this peculiarity can be found in the relationship that, according to Jaggar, exists between outlaw emotions and critical social theory, where the latter is understood as the development of collective, and eventually emancipatory knowledge. This relationship’s peculiarity lies in the fact that outlaw emotions seem to be necessary for the production of critical theory (feminist consciousness, subculture) but at the same time seem to “presuppose at least the beginning of such a perspective” (Jaggar, 1989, p. 177), making it difficult to understand where one begins and the other ends, or rather, to stay with the “the radical feminist metaphor of the upward spiral” proposed by Jaggar (1989, p. 171), where it starts. In other words, outlaw emotions and critical theorizing stand in a “continuous feedback loop” (Jaggar, 1989, p.170).

In summary, the strength of the oppressed position seems to lie in two combined characteristics. The first is its dialectical character, exemplified in the moment of ‘suspension and hesitation’ of double ontological shock. This hesitation is addressed by scholars of the phenomenology of oppression, such as Fanon and Beauvoir, and is a fundamental opportunity to generate “possibility by articulating experience anew, interrupting its naturalizing tendencies and making experience hesitate” (Al-Saji, 2017, p. 152). In phenomenological terms, this strength of the oppressed position, which standpoint theory calls epistemic privilege, lies in its ambiguity. The second feature is the interpersonal, collective, and achieved character of the oppressed position, that is, its need to be shared and validated by others. The intersubjective nature of this diagnostic process is crucial: it is in relationship with others that allows us to move from individual symptoms to collective diagnosis.

### 3.4 Ambiguity, or the world as “revealed only through rejection, desire, hate and love”

Simone de Beauvoir’s *Ethics of Ambiguity* offers valuable tools for understanding what happens in the process of changing oppressive beliefs. Her analysis of ambiguity illuminates the relationship between transcendence, freedom, and emotions, providing an interpretive key for the “double ontological shock” described by Bartky.

For Beauvoir, ambiguity fundamentally lies in a set of human conditions that are universal and insurmountable: human mortality and human awareness of mortality; the human awareness of being a subject and, at the same time of being an object for others, of being objectified by others, an object between other objects in the world; and the experience of being an individual and at the same time a fundamental part of the collective — and necessarily dependent on it. This ambiguity can also be framed as the impossibility or rejection of adhering to what is, or: ambiguity is aspiring to transcendence. Human beings aspire to transcendence. Bartky (1975, p. 429), building on Sartre’s work, individuates in a feminist’s transcendence the “project of negation and transformation” that “*makes possible what are specifically feminist ways of apprehending contradictions in the social order.”*

Transcendence is a significant though not exclusive attribute of the oppressed (inclusive of double ontological shock). Human beings share this aspiration toward transcendence, and they are conscious that other human beings have the same aspiration. However, the frustration that emerges from this condition does not concern every person in the same way. In fact, it concerns particularly those who are subjected to a situation of oppression. On oppression, Beauvoir writes:

*(…) every man transcends himself. But it happens that this transcendence is condemned to fall uselessly back upon itself because it is cut off from its goals. That is what defines a situation of oppression. Such a situation is never natural: man is never oppressed by things (…)* (Beauvoir, 1947, p. 87)

In Beauvoir’s formulation, humans pursue transcendence through the goals or projects that one decides and chooses for himself, and oppression is defined by ‘being cut off” from this possibility. In other words, being oppressed is being restricted to a condition in which one cannot transcend oneself, in fact, is relegated to the contingency of what is. Every human being is part of the world, situated in the world, and either oppressed or oppressing someone else (Beauvoir, 1947, pp. 83–84).^14^ Moreover, Beauvoir notes that ‘man is never oppressed by things,” that is, oppression is always human-made.^15^

Emotions related to oppression have an important role in Beauvoir’s theory. She addresses the topic at the beginning of the section “The Aesthetic Attitude,” where she discusses the distinction between past and present, and tackles the different attitudes we should adopt regarding the past, and regarding the present. While one can look at the past with an attitude of ‘joyful contemplation’, the attitude towards the present cannot be contemplative:^16^ one should not consider with impartial interest the things that exist today. Doing so regarding the present is an attitude that ‘appears in moments of discouragement and confusion”— it is a ‘withdrawal’ position, “a way of fleeing the truth of the present’ (Beauvoir, 1947, p. 81). Contemplation can, however, be legitimate toward the past, and the past world may seem justified (also because it can be transfigured by artistic creation). But contemplation is not a legitimate attitude towards the present: the present ‘is the moment of choice and action,’^17^ it is the moment of the *project*. Contemplation is undesirable toward the present, and the present world can be revealed only through our interaction with it. This interaction displays emotional features:

*If we first considered the world as an object to be manifested, if we thought that it was saved by this destination in such a way that everything about it already seemed justified and that there was no more of it to reject, then there would also be nothing to say about it, for no form would take shape in it; it is revealed only through rejection, desire, hate and love* (Beauvoir, 1947, p. 83).^18^

The takeaway from this passage is that the world can “be revealed only through rejection, desire, hate, and love.” It is from this relationship with the world—a relationship mediated by emotions—that the desire for freedom for oneself *and* for others unfolds.

In summary, Beauvoir suggests that the project of freedom is to disclose; human beings disclose, and disclosing is possible through the experience of emotions (not through the attitude of contemplation). Disclosure is crucial in Beauvoir’s philosophy since, as she explains, it is the same as desiring freedom: “To will freedom and to will to disclose being are one and the same choice” (Beauvoir, 1947, p. 84). Double ontological shock occurs when the human being, who desires freedom *and* disclosure, is hindered by oppression. Therefore, he^19^ feels rejection, desire, hate, and love. This is the moment in which emotions shape the emancipation of the individual. For Beauvoir, action and revolt are necessary from here to the next step. In revolt, the human ‘prove[s] that he is a man and that he is free’ (Beauvoir, 1947, p. 89).^20^ Revolt has its roots in the possibility of approaching reality with an attitude that is fueled by emotions, avoiding contemplation and instead choosing to act on these emotions. The input of this action is the opposite of the contemplative way, that is, the possibility of feeling emotions towards the present reality. Indeed, emotions play this relevant role for every human being, but more so for the oppressed, who cannot accept being deprived of the possibility of transcendence, that is, to will freedom both for themselves and for others. If the contemplative attitude offers a viable possibility for people who are not oppressed, the same is not true for oppressed human beings, who need to revolt to pursue their own transcendence.

Beauvoir’s analysis of emotions as revelatory (‘it is revealed only through rejection, desire, hate and love’) offers a phenomenological perspective that complements Jaggar’s epistemological approach to “outlaw emotions.” Both thinkers recognize that emotions, particularly in contexts of oppression, are not simple passive reactions but active ways of relating to the world that can motivate transformation and transcendence. This convergence between phenomenology and feminist epistemology confirms our first methodological thesis that calls for an integration of these approaches in the study of emotions.

## 4 Conclusion

The path articulated thus far between phenomenology and feminist epistemology has elucidated several fundamental aspects of emotions. Emotions are crucial players in knowledge processes, serving as diagnostic tools for what Alia Al-Saji calls “pathologies of the social.” They signal what matters to us and speak to the relational qualities that connect us to the world and to others.

This relationship with the world is one of co-implication, where we are not detached observers but rather fundamentally entangled with our environment. This co-implication becomes more perceptible and visible in extreme cases related to oppression, where we more acutely perceive the friction in our relationship with the world. Double ontological shock exemplifies this friction, revealing how emotions can function both as symptoms of oppression and as catalysts for transformation.

Particularly significant in this relationship of co-implication is the dimension of our connection with others, where the friction is expressed and dialectically re-elaborated—a process captured in Jaggar’s metaphor of the “upward spiral.” It is in relationship with others (whom we perceive as free, as outlined in Beauvoir’s analysis) that we find the prerequisites for our own liberation. As Beauvoir suggests, our encounter with the world “is revealed only through rejection, desire, hate and love,” and it is precisely through these emotional engagements that we can disclose being and will freedom for ourselves and others.

The analysis of oppression-related emotions thus reveals broader ontological characteristics of emotions: they are embodied and situated, necessarily intersubjective, action-oriented, and capable of transcending oppressive structures while still being conditioned by them. By understanding emotions not as mere obstacles to knowledge but as sophisticated modes of disclosing reality, particularly in contexts of oppression, we gain valuable insights into both their epistemological significance and their potential role in emancipatory practices.

These ontological characteristics of emotions can be generalized beyond contexts of oppression. Emotions universally function as modes of co-implication with the world, attuned to relational qualities that matter to us. Their diagnostic capacity lies precisely in this attunement: emotions detect fissures in our expected relationships with environments, others, and ourselves before they can be conceptually articulated.

The understanding of emotions as diagnostic tools can inform various domains beyond the feminist context, including clinical practice, social policy, and educational interventions. In each of these contexts, attending to emotions – particularly those that seem out of place or dissonant – may reveal problems in social systems that have yet to be conceptually identified. Moreover, these findings open potential dialogues with contemporary theories of emotions. Slaby’s (2014) work on extended emotions, for example, explores how emotional processes can be constitutively extended by environmental and social structures, offering one possible framework for developing further the co-implicative relationship between emotions and world disclosed in our analysis. Similarly, the growing literature on second-person perspectives in emotion theory might provide additional insights into the intersubjective dimensions of emotions revealed in contexts of collective resistance to oppression.

The diagnostic framework developed here invites further research on the epistemological status of emotional evidence, the relationship between individual emotional symptoms and collective diagnoses, and the development of practices that facilitate the articulation of emotional diagnoses into socially transformative action. Reconceptualizing emotions as sophisticated diagnostic instruments rather than mere reactions contributes to a more comprehensive understanding of how knowledge about social reality is produced and how social change becomes possible. The integration of phenomenology and feminist epistemology in the study of emotions uncovers new avenues for research, particularly regarding the collective validation and transformation of emotional insights into critical knowledge, the role of emotions in alternative relational ontologies, and their implications for education and social change oriented toward liberation.

## Footnotes

^1^In this article, the term ‘emotion’ is used as an “umbrella-term, encompassing a vast range of diverse affective phenomena” (Szanto and Landweer, 2020, p. 1). While having different concepts for affect and emotion (as outlined, for example, in Massumi 2002, p. 28) may be useful in some circumstances, it is not necessary for the scope of this article.

^2^With this term Al-Saji defines not so much the world’s structural injustices (or “oppression”) as their “pathological recalcitrance (…) in how they manage relationality, in their dependence on, forgetfulness of, and domination of others.” (Al-Saji, 2017, p. 150). Relationality is a theme that will be approached later in this article.

^3^“Feminist standpoint theory” is used here to refer to a strand, or family, of feminist epistemologies, methodologies and research that exhibits a rich internal heterogeneity, for which it is not really possible to postulate just *a* singular, or *the*, feminist standpoint. For example, the position of Black women, and therefore their feminist standpoint, can be very different from that of white women: one of the founders of feminist standpoint theory, Patricia Hill Collins, explored this issue in her work both as a standpoint theorist and as a founder of intersectionality (Collins, 1986, 2019). That said, the label has become ubiquitous after Sandra Harding used it to define one of the three strands in feminist epistemology described in her classic monograph *The Science Question in Feminism* (Harding, 1986). Even if Harding’s tripartition in feminist empiricism, standpoint theory, and postmodernism is now considered insufficient to describe the complexity of the feminist epistemologies field (Tanesini, 2015), “feminist standpoint theory” is still consistently used, in research and in philosophical literature, to indicate theories that share some ideas or unifying characters (discussed more below, in section 2 and 3.2). One of the most relevant among them is the indication or prescription of starting the inquiry from marginalized groups’ experience and their understanding of their experience, because in that understanding something can be found that cannot be found elsewhere, that is, their positioning allows them to have an “epistemic privilege” (for more see more Par. 3.2). I thank Reviewer 1 for pointing out that an acknowledgement of the richness and heterogeneity of standpoint theory was due, and that my previous formulation was at risk of erasing the role of Black feminist thought in standpoint theory. I would like to emphasize that I consider the latter not ‘a contribution’ external to the feminist tradition, but one of the founding feminist “theorizing” that brought to life feminist epistemology itself [I use here “theorizing” referring to Collins’ use of the term, see for example Collins (2019, pp. 12, 88)].

^4^“Giving a damn, having something matter, genuine caring for something or someone—that is an excellent attempt at capturing, in a nutshell, something like the “essence” of emotion, or rather: of emotionality as the general capacity for experiencing distinct emotional states or processes” (Slaby, 2014, p. 32).

^5^See for example the entry “Emotion” in the Stanford Encyclopedia of Philosophy: “In view of the proliferation of exchanges between researchers of different stripes, it is no longer useful to speak of the philosophy of emotion in isolation from the approaches of other disciplines, particularly psychology, neuroscience, and evolutionary biology. This is why we have made an effort to pay significant attention to scientific developments, as we are convinced that cross-disciplinary fertilization is our best chance for making progress in emotion theory” (Scarantino and de Sousa 2021).

^6^Another naturalized and non-reductionist theory of emotion that is part of the theoretical background of this article, even if it cannot be properly addressed in this context, is that of Hufendiek (2016, 2017, 2021), although it should be noted that it is not explicitly feminist.

^7^In this regard, it would be interesting to understand whether Husserl’s and, more generally, phenomenology’s critique of naturalism is also relevant to feminist naturalism, or, conversely, whether it is even possible to speak of a *naturalized* feminist philosophy. If the main problem of naturalism is as stated by Carel and Meacham (2013), commenting on Dermot (2013), that “Naturalism misunderstands the world because it misunderstands the subject’s necessary role in the project of knowledge, and in the very constitution of objectivity. Naturalism subtracts the knowing subject from the process of knowledge, and then treats the desiccated product as if it were the real world.” (Carel and Meacham, 2013, Dermot, 2013, ‘“Let us Look at It Objectively”: Why Phenomenology Cannot be Naturalized’), then feminist epistemologies, which focus on the necessity of the subjects’ role and agency for objectivity, may be excluded from this critique, or may be not consider naturalized at all from a phenomenological perspective.

^8^“While giving a “phenomenology of x” is sometimes used loosely to mean describing what it is like to experience or be x, in the first person, this is not all that phenomenology as method must do. Phenomenology both makes experience (partially) explicit and discloses that which is structuring of, which makes a difference in, experience. It gestures toward that which is only indirectly and laterally given in experience—the invisible norms according to which meaning appears. Such normativity—of perception, for instance—is historically instituted and socially situated, but it is forgotten as norm and its work remains invisible” (Al-Saji, 2017, p. 149).

^9^This article focuses on the phenomenological and epistemological aspects of the thorny problem, but a few words about the metaphysical problem are in order. On the metaphysical side, the thorny problem raises the question of what exceeds, transcends, or resists the constructed oppression that informs the subject. This metaphysical question is important and problematic for multiple frameworks, since all social constructionist frameworks ultimately raise the question of what resists oppression.

^10^The first example may remind readers of the problem of ‘testimonial injustice,’ while the second may remind them of ‘hermeneutical injustice.’ These labels belong to the framework of ‘epistemic injustice’ (first conceptualized by Fricker, 2007), influential in the contemporary philosophical debate. While I am aware that these topics are discussed in the epistemic injustice debate, in this article I resist using them to remain with language more suitable to my preferred frameworks, and therefore of the sources here investigated (feminist standpoint theory, feminist phenomenology, and feminist new materialism). This is not the only reason to resist the epistemic injustice framework: for a compelling critique of the latter, see Crary (2018).

^11^Specifically, it focuses on the psychological experience of oppressed individuals, as was initially explored in the work of Franz Fanon.

^12^However, Jaggar does not use these names to refer to the experience.

^13^I use “situated knowledge” thesis here to utilize Toole’s nomenclature, which in turn recalls Donna Haraway’s essay, “Situated Knowledges: The Science Question in Feminism and the Privilege of Partial Perspective” (1988). Haraway’s essay is a crucial reference for the positioning or situated knowledges thesis, but it is not (or, at least, not entirely) a standpoint theory text. Instead, it interacts with that tradition: its interlocutors are feminist standpoint and empiricist theorists; and it has been integrated in later feminist standpoint work; therefore, is often referred to as a standpoint theory source.

^14^Every human being must desire freedom for both self and others. Freedom, here, is simply understood as the absence of oppression. Every project must be evaluated in relation to this freedom. Also, Beauvoir (1947, p. 89) reiterates, “the world is divided into two clans, oppressed and oppressors.” Here, the reference is to the class axis, but it can also be applied to gender (including in relation to reproductive labor).

^15^From this claim it can be inferred that one cannot define “oppression” as something that just happens to be: if one is born without a limb, that is not the cause of their oppression. The cause of their oppression are the systems made by other humans. An earthquake, in itself, is not oppressive. A flood that is the result of climate change, which is caused by humans, is an effect of oppression. To expound Beauvoir’s concept of oppression in posthumanist terms, animals may also be affected by oppression, if we accept that animals may have their own transcendent goals and that human beings limit their ability to pursue them.

^16^The contemplative mode described by Beauvoir share similarities with the notorious problem of the detached gaze in feminist epistemologies. The traditional reference for this problem is Haraway (1988). While certainly the detached gaze and a joyful contemplation do not seamlessly overlap, there can be a joy in claiming the detached gaze, a joy that is not accessible to *all* subjects and may indeed have disturbing implications with respect to the overpowering of positionings that self-disclose as non-detached.

^17^Beauvoir (1947, p. 82).

^18^This reasoning is intimately connected with Beauvoir’s reflection on the role of art and of the artist, but in this article the aesthetic aspect is deliberately neglected to focus on the role of emotions at this juncture.

^19^To stay faithful to the text, I use the same pronoun here that Beauvoir uses in the passage quoted earlier; but of course, the subject of this example may be of any gender.

^20^Beauvoir continues: “The oppressed has only one solution: to deny the harmony of that mankind from which an attempt is made to exclude him, to prove that he is a man and that he is free by revolting against his tyrants. In order to prevent this revolt, one of the rushes of oppression is to camouflage itself behind a natural situation since, after all, one can not revolt against nature.” The topic of revolt against what is passed off as nature is another major theme in feminist literature, but it is beyond the scope of this article.
